# Optimized Projection and Fisher Discriminative Dictionary Learning for EEG Emotion Recognition

**DOI:** 10.3389/fpsyg.2021.705528

**Published:** 2021-06-28

**Authors:** Xiaoqing Gu, Yiqing Fan, Jie Zhou, Jiaqun Zhu

**Affiliations:** ^1^School of Computer Science and Artificial Intelligence, Changzhou University, Changzhou, China; ^2^Viterbi School of Engineering, University of Southern California, Los Angeles, CA, United States; ^3^School of Electrical and Mechanical Engineering, Shaoxing University, Shaoxing, China

**Keywords:** EEG signal, emotion recognition, dictionary learning, fisher discrimination criterion, brain computer interface

## Abstract

Electroencephalogram (EEG)-based emotion recognition (ER) has drawn increasing attention in the brain–computer interface (BCI) due to its great potentials in human–machine interaction applications. According to the characteristics of rhythms, EEG signals usually can be divided into several different frequency bands. Most existing methods concatenate multiple frequency band features together and treat them as a single feature vector. However, it is often difficult to utilize band-specific information in this way. In this study, an optimized projection and Fisher discriminative dictionary learning (OPFDDL) model is proposed to efficiently exploit the specific discriminative information of each frequency band. Using subspace projection technology, EEG signals of all frequency bands are projected into a subspace. The shared dictionary is learned in the projection subspace such that the specific discriminative information of each frequency band can be utilized efficiently, and simultaneously, the shared discriminative information among multiple bands can be preserved. In particular, the Fisher discrimination criterion is imposed on the atoms to minimize within-class sparse reconstruction error and maximize between-class sparse reconstruction error. Then, an alternating optimization algorithm is developed to obtain the optimal solution for the projection matrix and the dictionary. Experimental results on two EEG-based ER datasets show that this model can achieve remarkable results and demonstrate its effectiveness.

## Introduction

Brain–computer interface (BCI) has been one of the research hotspots in recent years in health monitoring and biomedicine (Edgar et al., 2020; Ni et al., 2020b). The BCI does not rely on muscles and the peripheral nervous system. It establishes a direct information transmission channel between the brain and the outside world. The electroencephalography (EEG) signals captured by the BCI system are a powerful tool to analyze neural activities and brain conditions. EEG has the advantages of convenience (i.e. non-invasive, non-destructive and simple) and validity (i.e. sensitivity, validity and compatibility) (Sreeja and Himanshu, 2020). EEG signal is an important tool for revealing the emotional state of human beings. It has been shown that when people are in different thinking and emotional states, the rhythm components of EEG signals are different from their waveform. In BCI, the operation of emotion recognition (ER) starts from external stimuli to subjects, which induce specific emotions such as happiness, sadness, and anger. These stimuli may be videos, images, music, and so on. During the session, EEG data are recorded by EEG devices. Subsequently, the first step is to extract and preprocess useful features obtained from the recorded EEG. The next step is to train the classifier and optimize the parameters. The final step is to test the training model with new EEG data that are not used in the training process.

Traditional machine learning classifiers have been widely used in EEG-based ER, such as support vector machine (SVM) (Zheng et al., 2019), deep learning (Hwang et al., 2020; Song et al., 2020), nearest neighbor classifier (Li et al., 2019), random forest (Fraiwan et al., 2012), and probabilistic neural networks (Nakisa et al., 2018). In recent years, dictionary learning-based methods have achieved great success in EEG-based recognition tasks for BCI (Ameri et al., 2016; Gu et al., 2020; Ni et al., 2020a). In general, dictionary learning-based classification methods often learn the discriminative and robust dictionaries from training samples. The test sample is sparsely represented as a sparse linear combination of atoms by the learned dictionary, and then, the classification task can be carried out according to the reconstruction error and/or the sparse coefficients. Dictionary learning works well-even with noisy EEG signals. Barthélemy et al. (2013) developed an efficient method to represent EEG signals based on the adapted Gabor dictionary and demonstrated on real data that the learned multivariate model is flexible and the learned representation is informative and interpretable. Abolghasemi and Ferdowsi (2015) developed a dictionary learning framework to remove ballistocardiogram (BCG) artifacts from EEG. Given the advantage of the noise-robust sparse dictionary, a new cost function was proposed, which can model BCG artifacts and then remove them from the original EEG signals. Kashefpoor et al. (2019) developed a correlational label consistent K-SVD dictionary learning method applied to EEG-based screening tool. This method was applied to speckle extraction of EEG signals and extracted spectral features in both time and frequency domains. Aiming at the problem that eye movement and blinking can cause artifacts, Kanoga et al. (2019) proposed a multi-scale dictionary learning method to eliminate eye artifacts from single-channel measurement. Specifically, the time-domain waveforms related to repetitive phase events in EEG signals were learned within the framework of dictionary learning. And the proposed multi-scale dictionary learning method was used to represent the signal components on different timescales. To achieve the highly accurate classification of EEG in BCI, Huang et al. (2020) developed a signal identification model using sparse representation and fast compressed residual convolutional neural networks (CNNs). The authors used the common spatial patterns to extract EEG signal features and build a redundant dictionary using these features. Then, the proposed deep model as a classifier recognized the input EEG signals.

Although machine learning has achieved good classification performance in some application scenarios, the accuracy and applicability of the classification do not go far enough. Since EEG data provide comprehensive information across different frequency bands to characterize emotions, it was expected to design an ER method, which utilizes the specific discriminative information of each frequency band and preserves the common discrimination information shared by multiple band signals. After the success of dictionary learning, in this study, we propose optimized projection and Fisher discriminative dictionary learning (DDL) for EEG-based ER. According to the Fisher discrimination criterion of minimum within-class sparse reconstruction error and maximum between-class sparse reconstruction error, we learn the discriminative projection to map the multiple band signals into a shared subspace and simultaneously build a shared dictionary that establishes the connection between different bands and represents the characteristics of signals well. Therefore, the joint learning of projection and dictionary ensures the common internal structure of multiple frequency bands of signals to be mined in the subspace.

The main contributions of this study are as follows:

(1) A multiple frequency band collaborative learning is introduced in dictionary learning for the EEG-based ER. This learning mechanism can efficiently integrate the band-independent information and inter-band correlation information.
(2) Through the feature projection matrix, the data of multiple frequency bands are projected into a common projection subspace to keep the latent manifold of EEG signals. Meanwhile, the discriminative dictionary is learned by enforcing the classification criterion so that the learned sparse code has a strong representation and discrimination ability.
(3) This joint optimization method has some benefits. Learning independent projection matrices makes this model easily extensible; meanwhile, learning a dictionary in a subspace allows abandoning extraneous information in the original features. In addition, the alternating optimization procedure ensures the dictionary and projection are optimized at the same time.
(4) These extensive experiments on the SEED and DREAMER datasets demonstrate that the multiple band collaborative learning is effective, and this method can improve the discrimination ability of sparse coding in EEG-based ER.

## Background

### Datasets

The experimental data in this study are taken from two public EEG emotion datasets: SEED (Zheng and Lu, 2015) and DREAMER (Katsigiannis and Ramzan, 2018). Table 1 briefly describes the information of the two datasets. Both SEED and DREAMER datasets are collected when subjects watched emotion-eliciting movies. In the SEED dataset, each subject participated in three experiments, which were separated into three time periods, corresponding to three sessions, and each session corresponds to 15 EEG data trials. Thus, a total of 15 × 3 = 45 trials are formed per subject. The SEED provided five frequency bands: δ band (1–3 Hz), θ band (4–7 Hz), α band (8–13 Hz), β band (14–30 Hz), and γ band (31–50 Hz). For the DREAMER dataset, the data recorded by each subject contain three parts: 18 experimental signal segments, 18 baseline signal segments corresponding to relaxation state, and 18 corresponding labels. The DREAMER data provided EEG features with frequency bands θ, α, and β.

**Table 1:** The basic information of SEED and DREAMER datasets.

**SEED dataset**	**DREAMER dataset**
15 subjects	23 subjects
15 trials using Chinese movie clips (length of film clips about 4 min) to evoke emotions as negative, positive, and neutral	18 trials using movie clips (length of film clips 65–393 s) to evoke 9 emotions
In one session, 5 s hint before each clip, 45 s self-assessment, and 15 s rest after each clip	At least 61 s of pretrial baseline data available
Emotion rating metric: negative, positive, and neutral	Emotion rating scale: valence, arousal, and dominance on a continuous scale from 1 to 5
62-channel electrode cap	14-channel electrode cap
Sampling rate 1,000 Hz	Sampling rate 128 Hz
Frequency band from 0 to 75 Hz	Frequency band from 4 to 30 Hz

### Machine Learning-Based EEG Signal Processing Program

**Figure 1:**
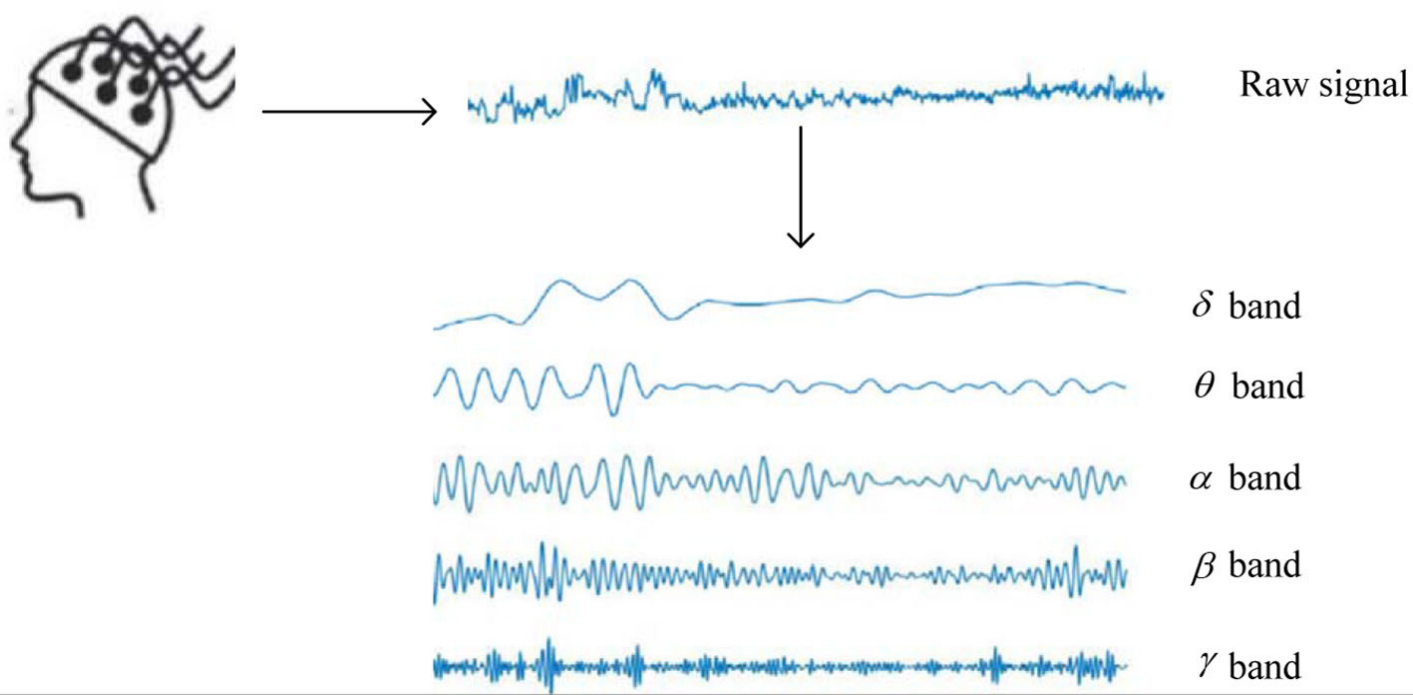
The process of constructing multiple frequency band sequences (Wei et al., 2020).

For machine learning-based EEG-based ER, feature extraction and emotion classification are the critical procedures. Considering the SEED dataset as an example, the process of constructing five frequency band sequences is described in Figure 1. Firstly, EEG signals collected by BCI are preprocessed by filtering. Then, according to the characteristics of different rhythms of EEG signals, EEG signals usually can be divided into several rhythmic signal components ranging from 0 to 50 Hz. Secondly, EEG features can be extracted by various strategies. Time-domain, frequency-domain, and non-linear analysis methods are the three types of most commonly used EEG feature extraction methods. The time-domain features aim to capture the temporal information of EEG signals, such as higher-order crossings (HOC) (Petrantonakis and Hadjileontiadis, 2010), Hjorth features (Petrantonakis and Hadjileontiadis, 2010), and event-related potential (ERP) (Brouwer et al., 2015). The frequency-domain features aim to capture primarily the EEG emotion information from a frequency perspective. Then, EEG features can be extracted by various methods, such as rhythm (Bhatti et al., 2016), wavelet packet decomposition (WPD) (Wu et al., 2008), and approximate entropy (AE) (Ko et al., 2009). Non-linear features are extracted from the transformed phase space. Non-linear features contain quantitative measures that represent the complex dynamic characteristics of the EEG signals, such as Lyapunov exponent (Lyap) (Kutepov et al., 2020) and correlation dimension (CorrDim) (Geng et al., 2011). Finally, many machine learning methods are established to handle EEG emotion classification on extracted feature sets.

A common approach to deal with multiple bands of EEG data using traditional dictionary learning methods is to directly concatenate features of multiple bands together in the high-dimensional space and treat this single feature vector as the input to the model. However, dictionary learning may not perform well because different band features usually carry different characteristics of EEG emotion.

### Dictionary Learning

Let $\text{X}=\left[{\text{x}}_{1},...,{\text{x}}_{n}\right]\in {\text{R}}^{m\times n}$ be a set of *m*-dimensional *n* training signals. To minimize the reconstruction error and satisfy the sparsity constraints, the sparse representation and dictionary learning of **X** can be accomplished by

(1) $$\begin{array}{l} \begin{array}{l} \underset{\text{D},\text{S}}{\min}\|\text{X}-\text{D}\text{S}{\|}_{F}^{2},\\ s.t.\;{\left\|{\text{s}}_{i}\right\|}_{0}\leq T, \end{array} \end{array}$$

where $\text{D}=\left[{\text{d}}_{1},...,{\text{d}}_{K}\right]\in {\text{R}}^{m\times K}$ is a dictionary with *K* atoms $\text{S}=\left[{\text{s}}_{1},...,{\text{s}}_{n}\right]\in {\text{R}}^{m\times K}$ is the sparse coefficient matrix of signals **X**, and **s**_*i*_ is the sparse coefficient vector of **x**_*i*_ over **D**. *T* is the sparse constraint factor. The ||_**s**_*i*_||0_ ≤ *T* term requires the signal **x**_*i*_ to have fewer than *T* non-zero items in its decomposition.

It is not easy to find the optimal sparse solution using ℓ_0_-norm regularization term; thus, an alternative formulation of Equation (1) is to replace it with ℓ_1_-norm regularization as

(2) $$\begin{array}{l} \begin{array}{l} \underset{\text{D},\text{S}}{\min}\|\text{X}-\text{D}\text{S}{\|}_{F}^{2}+\lambda {\left\|\text{S}\right\|}_{1},\\ s.t.\;\|{\text{d}}_{i}{\|}_{2}^{2}=1,\forall i \end{array}, \end{array}$$

Equation (2) can be optimized by many efficient ℓ_1_ optimization methods, such as the famous K-SVD algorithm (Aharon et al., 2006; Jiang et al., 2013). However, Equation (2) is an unsupervised learning framework. To learn a discriminative dictionary for classification tasks, different kinds of loss functions or Fisher discrimination criterion are considered in the dictionary learning. Fisher discrimination constraints on atoms of the dictionary (Peng et al., 2020) or sparse coefficient **S** (Li et al., 2013) or reconstruction error of **X** (Zheng and Sun, 2019; Zhang et al., 2021) strive to preserve the class distribution and geometric structure of data.

Suppose data matrix **X** consists of samples from *C* different classes, from **X**, both the sub-dictionary **D**_*i*_ and sub-sparse coefficient matrix **S**_*i*_ are learned for the *i*-th class data (*i* = 1, 2,.,*C*). The whole dictionary **D** is represented as **D** = [**D**_1_, **D**_2_, ..., **D**_*C*_]. Let ${\text{W}}_{w}^{\;}$ and ${\text{W}}_{b}^{\;}$ denote the within-class scatter and between-class reconstruction error of **X**, respectively, then

(3) $$\begin{array}{l} {\text{W}}_{w}^{\;}=\underset{j}{\sum }\left({\text{x}}_{j}^{\;}\text{-}\text{D}\delta_{l_{j}}\left({\text{s}}_{j}^{\;}\right)\right)\times {\left({\text{x}}_{j}^{\;}\text{-}\text{D}\delta_{l_{j}}\left({\text{s}}_{j}^{\;}\right)\right)}^{T}, \end{array}$$

and

(4) $$\begin{array}{l} {\text{W}}_{b}^{\;}=\overset{\;}{\underset{j}{\sum }}\left({\text{x}}_{j}^{\;}\text{-}\text{D}\zeta_{l_{j}}\left({\text{s}}_{j}^{\;}\right)\right)\times {\left({\text{x}}_{j}^{\;}\text{-}\text{D}\zeta_{l_{j}}\left({\text{s}}_{j}^{\;}\right)\right)}^{T}, \end{array}$$

where δ_*l*_*j*__( ) function returns the sparse codes consistent with the class of ${\text{x}}_{j}^{\;}$ (*j* = 1,2,.,*n*), and ζ_*l*_*j*__( ) function returns the sparse codes not consistent with the class of ${\text{x}}_{j}^{\;}$.

Then, a discriminative dictionary can be learned by reducing the within-class diversity and by increase between-class separation using Equations (3) and (4) (Zheng and Sun, 2019; Zhang et al., 2021).

## Optimized Projection and Fisher Discriminative Dictionary Learning

### Objective Function

**Figure 2:**
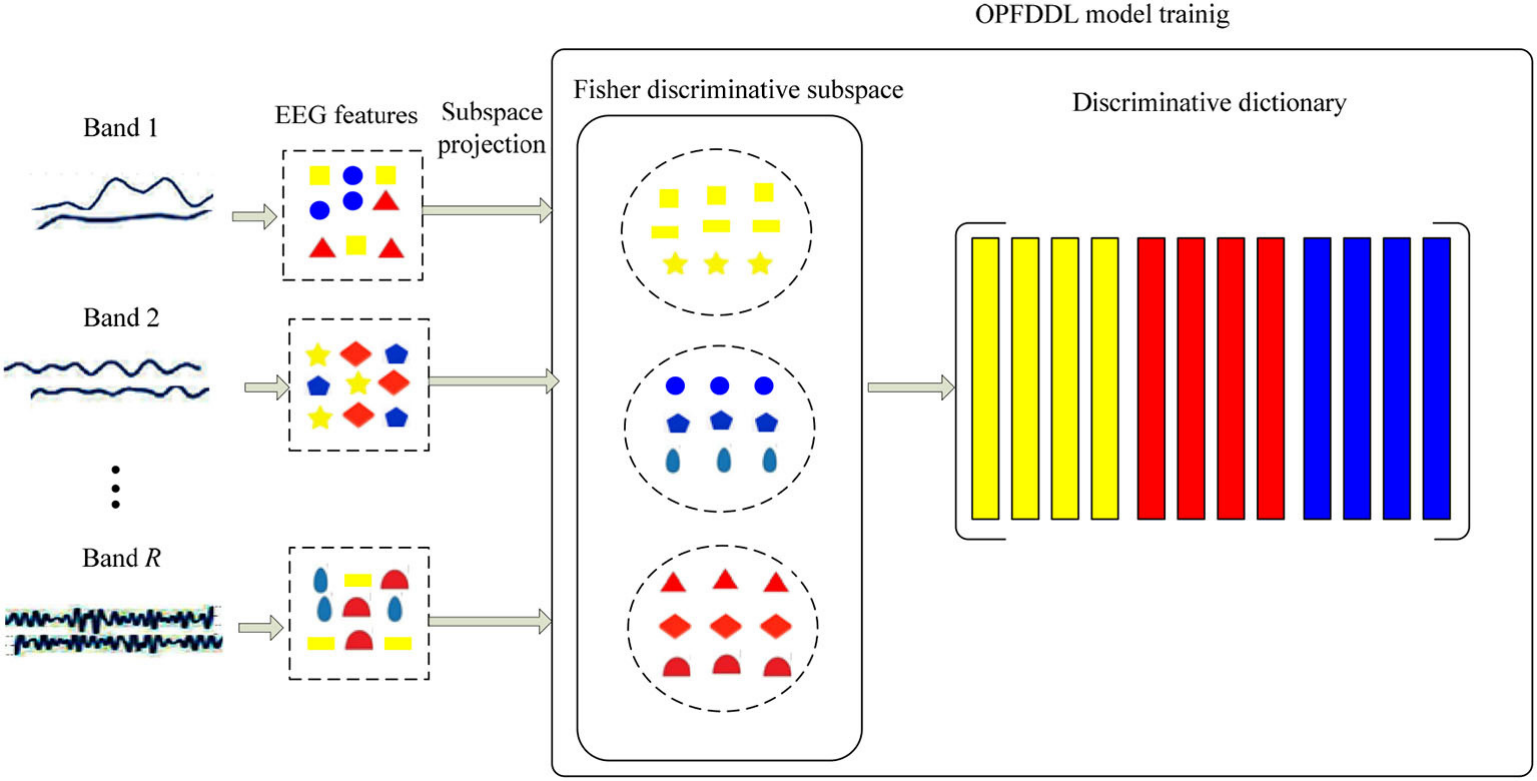
The training framework of optimized projection and Fisher discriminative dictionary learning (OPFDDL) model.

Here, we describe in detail the optimized projection and Fisher DDL model for collaborative learning of multiple frequency band EEG signals. The training framework of the OPFDDL model is illustrated in Figure 2. We learn a discriminative projection to map multiple frequency band EEG signals into a common subspace; simultaneously, we learn a common discriminative dictionary to encode the band-invariant information of multiple frequency bands. In particular, to promote the discrimination ability of the model, we utilize the model according to the Fisher discrimination criterion (Gong et al., 2019) under the structure of dictionary learning.

Let ${\text{X}}^{r}=\left\{{\text{x}}_{j}^{r}\right\}$denote the signal set **X** of the frequency band *r*, where *R* is the number of frequency bands (*r* =1,., *R*). ${\text{x}}_{j}^{r}$ is the *j*th sample in **X**^*r*^. To build the connection between different frequency bands and exploit the specific characteristic of each representation, we project ${\text{x}}_{j}^{r}$ into a feature subspace as ${\text{z}}_{j}^{r}={\text{Q}}^{r}{\text{x}}_{j}^{r}$ by using a transformation matrix **Q**^*r*^ ∈ **R**^*m* × *d*^^*r*^. Therefore, we obtain ${\left\{{\text{z}}_{j}^{r}\right\}}_{r=1}^{R}$ by ${\left\{{\text{Q}}^{r}{\text{x}}_{j}^{r}\right\}}_{r=1}^{R}$ as the feature representations for *R* frequency bands. Then, we denote the within-class reconstruction error $J_{w}^{r}$ and between-class reconstruction error $J_{b}^{r}$ of the *r*th frequency band in the projection subspace

(5) $$\begin{array}{l} \begin{array}{l} J_{w}^{r}=tr\left(\overset{n_{r}}{\underset{j=1}{\sum }}\left({{\text{Q}}^{r}}^{T}{\text{x}}_{j}^{r}\text{-}{{\text{Q}}^{r}}^{T}\text{D}\delta_{l_{j}}\left({\text{s}}_{j}^{r}\right)\right)\times {\left({{\text{Q}}^{r}}^{T}{\text{x}}_{j}^{r}\text{-}{{\text{Q}}^{r}}^{T}\text{D}\delta_{l_{j}}\left({\text{s}}_{j}^{r}\right)\right)}^{T}\right)\\ =tr\left({{\text{Q}}^{r}}^{T}\overset{n_{r}}{\underset{j=1}{\sum }}\left({\text{x}}_{j}^{r}\text{-}\text{D}\delta_{l_{j}}\left({\text{s}}_{j}^{r}\right)\right)\times \left({\text{x}}_{j}^{r}\text{-}\text{D}\delta_{l_{j}}\left({\text{s}}_{j}^{r}\right)\right){\text{Q}}^{r}\right)\\ =tr\left({{\text{Q}}^{r}}^{T}{\text{W}}_{w}^{r}{\text{Q}}^{r}\right) \end{array}, \end{array}$$

and

(6) $$\begin{array}{l} \begin{array}{l} J_{b}^{r}=tr\left(\overset{n_{r}}{\underset{j=1}{\sum }}\left({{\text{Q}}^{r}}^{T}{\text{x}}_{j}^{r}\text{-}{{\text{Q}}^{r}}^{T}\text{D}\zeta_{l_{j}}\left({\text{s}}_{j}^{r}\right)\right)\times {\left({{\text{Q}}^{r}}^{T}{\text{x}}_{j}^{r}\text{-}{{\text{Q}}^{r}}^{T}\text{D}\zeta_{l_{j}}\left({\text{s}}_{j}^{r}\right)\right)}^{T}\right)\\ =tr\left({{\text{Q}}^{r}}^{T}\overset{n_{r}}{\underset{j=1}{\sum }}\left({\text{x}}_{j}^{r}\text{-}\text{D}\zeta_{l_{j}}\left({\text{s}}_{j}^{r}\right)\right)\times \left({\text{x}}_{j}^{r}\text{-}\text{D}\zeta_{l_{j}}\left({\text{s}}_{j}^{r}\right)\right){\text{Q}}^{r}\right)\\ =tr\left({{\text{Q}}^{r}}^{T}{\text{W}}_{b}^{r}{\text{Q}}^{r}\right) \end{array}, \end{array}$$

where ${\text{W}}_{w}^{r}=\overset{n_{r}}{\underset{j=1}{\sum }}\left({\text{x}}_{j}^{r}\text{-}\text{D}\delta_{l_{j}}\left({\text{s}}_{j}^{r}\right)\right)\times {\left({\text{x}}_{j}^{r}\text{-}\text{D}\delta_{l_{j}}\left({\text{s}}_{j}^{r}\right)\right)}^{T}$ is the within-class scatter matrix for sparse coding of the *r*th frequency band, and ${\text{W}}_{b}^{r}=\overset{n_{r}}{\underset{j=1}{\sum }}\left({\text{x}}_{j}^{r}\text{-}\text{D}\zeta_{l_{j}}\left({\text{s}}_{j}^{r}\right)\right)\times {\left({\text{x}}_{j}^{r}\text{-}\text{D}\zeta_{l_{j}}\left({\text{s}}_{j}^{r}\right)\right)}^{T}$ is the between-class scatter matrix for sparse coding of the *r*th frequency band.

From the classification point of view, minimizing within-class scatter and maximizing between-class scatter in the dictionary learning-based classifier can be represented as

(7) $$\begin{array}{l} \begin{array}{l} \underset{{\text{Q}}^{1},...,{\text{Q}}^{R},\text{D}}{\min}\frac{\overset{R}{\underset{r=1}{\sum }}tr\left({{\text{Q}}^{r}}^{T}{\text{W}}_{w}^{r}{\text{Q}}^{r}\right)}{\overset{R}{\underset{r=1}{\sum }}tr\left({{\text{Q}}^{r}}^{T}{\text{W}}_{b}^{r}{\text{Q}}^{r}\right)},\\ s.t.\;{\left({\text{Q}}^{r}\right)}^{T}\left({\text{Q}}^{r}\right)=\text{I},\;\forall r \end{array}, \end{array}$$

With definition as Q~ = [**Q**^1^, **Q**^2^, ..., **Q**^*R*^], ${\tilde{\text{W}}}_{w}=\left[\begin{array}{c} {\text{W}}_{w}^{1}\;\cdots \;0\\ \;\vdots \;\ddots \;\vdots \\ 0\;\cdots \;{\text{W}}_{w}^{R} \end{array}\right]$, and ${\tilde{\text{W}}}_{b}=[\begin{array}{c} {\text{W}}_{b}^{1}\;\cdots \;0\\ \;\vdots \;\ddots \;\vdots \\ 0\;\cdots \;{\text{W}}_{b}^{R} \end{array}],$

Equation (7) can be written as follows:

(8) $$\begin{array}{l} \begin{array}{l} \underset{\tilde{\text{Q}},\text{D}}{\min}\frac{tr\left({\tilde{\text{Q}}}^{T}{\tilde{\text{W}}}_{w}^{\;}\tilde{\text{Q}}\right)}{tr\left({\tilde{\text{Q}}}^{T}{\tilde{\text{W}}}_{b}^{\;}\tilde{\text{Q}}\right)},\\ s.t.\;{\left(\tilde{\text{Q}}\right)}^{T}\left(\tilde{\text{Q}}\right)=\text{I},\; \end{array} \end{array}$$

The projection matrix $\tilde{\text{Q}}$ is limited to be orthogonal, which is highly effective in the optimization process. The solution of Equation (8) refers to the complex inverse operation and is computationally intensive. Thus, we translate it into the following quadratic weighted optimization (QWO) problem and obtain the objective function of OPFDDL

(9) $$\begin{array}{l} \begin{array}{l} \underset{\tilde{\text{Q}},\text{D},\mu }{\min}\mu^{2}tr\left({\tilde{\text{Q}}}^{T}{\tilde{\text{W}}}_{w}^{\;}\tilde{\text{Q}}\right)-\mu tr\left({\tilde{\text{Q}}}^{T}{\tilde{\text{W}}}_{b}^{\;}\tilde{\text{Q}}\right),\\ s.t.\;{\tilde{\text{Q}}}^{T}\tilde{\text{Q}}=\text{I},\; \end{array}, \end{array}$$

It is noted that the parameter μ is an adaptive weight that can be obtained by a closed-form solution but is not a manually adjusted parameter.

### Optimization

In the following, the alternating optimization approach is used to update the parameters $\;\left\{\;\tilde{\text{Q}},\text{D},\mu \right\}$ in Equation (9).

**Figure 3:**
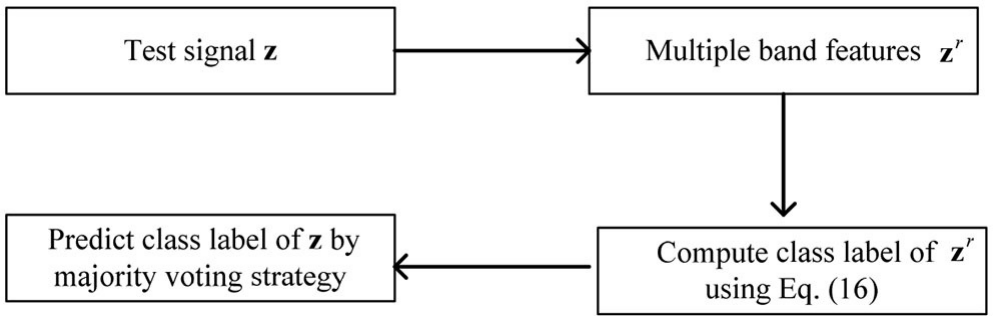
The testing procedure of OPFDDL.

(1) Update step for $\tilde{\text{Q}}$. With **D** and μ fixed, and with the known ${\tilde{\text{W}}}_{w}^{\;}$ and ${\tilde{\text{W}}}_{b}^{\;}$, the optimization of $\tilde{\text{Q}}$ can be solved by
  (10) $$\begin{array}{l} \left(\mu^{2}{\tilde{\text{W}}}_{w}^{\;}-\mu {\tilde{\text{W}}}_{b}^{\;}\right)\tilde{\text{Q}}=\gamma \tilde{\text{Q}}, \end{array}$$
  The projection matrix $\tilde{\text{Q}}$ is constituted by the feature vector corresponding to the first *d* minimum eigenvalues of Equation (10).
(2) Update step for **D**. With the definition of $\tilde{\text{X}}=\left[\begin{array}{c} {\text{X}}_{\;}^{1}\;\cdots \;0\\ \;\vdots \;\ddots \;\vdots \\ 0\;\cdots \;{\text{X}}_{\;}^{R} \end{array}\right]$, $\delta_{l_{j}}^{r}=\left[\delta_{l_{j}}\left({\text{s}}_{1}^{r}\right),\delta_{l_{j}}\left({\text{s}}_{2}^{r}\right),...,\delta_{l_{j}}\left({\text{s}}_{n_{r}}^{r}\right)\right]$, $\zeta_{l_{j}}^{r}=\left[\zeta_{l_{j}}\left({\text{s}}_{1}^{r}\right),\zeta_{l_{j}}\left({\text{s}}_{2}^{r}\right),...,\zeta_{l_{j}}\left({\text{s}}_{n_{r}}^{r}\right)\right]$, $\Omega =\left[\delta_{l_{j}}^{1},\delta_{l_{j}}^{2},...,\delta_{l_{j}}^{R}\right]$, and $\Theta =\left[\zeta_{l_{j}}^{1},\zeta_{l_{j}}^{2},...,\zeta_{l_{j}}^{R}\right]$, and with the known $\tilde{\text{Q}}$ and μ, Equation (9) can be written as
  (11) $$\begin{array}{l} \underset{D}{\max}\mu^{2}tr[({\tilde{Q}}^{T}\tilde{X}-{\tilde{Q}}^{T}D\Omega ){\left({\tilde{Q}}^{T}\tilde{X}-{\tilde{Q}}^{T}D\Omega \right)}^{T}]\\ -\mu tr[({\tilde{Q}}^{T}\tilde{X}-{\tilde{Q}}^{T}D\Theta ){\left({\tilde{Q}}^{T}\tilde{X}-{\tilde{Q}}^{T}D\Theta \right)}^{T}], \end{array}$$
  For each column of $\tilde{\text{X}}$, i.e., ${\tilde{\text{X}}}_{k}$, the optimization of **D** can be solved by the following problem:
  (12) $$\begin{array}{l} \frac{\partial L\left({\tilde{\text{X}}}_{k}\right)}{\partial \text{D}}=2\tilde{\text{Q}}{\tilde{\text{Q}}}^{T}\text{D}\left(\Omega \Omega^{T}+\Theta \Theta^{T}\right)-2\tilde{\text{Q}}{\tilde{\text{Q}}}^{T}{\tilde{\text{X}}}_{k}\left(\Omega^{T}+\Theta^{T}\right), \end{array}$$
  Then, ****D**** can be updated by
  (13) $$\begin{array}{l} \text{D}=\text{D}-\frac{\lambda_{D}}{n}\underset{k}{\sum }\frac{\partial L\left({\tilde{\text{X}}}_{k}\right)}{\partial \text{D}}, \end{array}$$
  where λ_*D*_ is the step size.
(3) Update step for **S**. When **D**, $\tilde{\text{Q}}$, and μ are learned, the sparse code **s** for each signal **x** in the subspace can be obtained by
  (14) $$\begin{array}{l} \underset{\text{s}}{\min}\|{\text{Q}}^{T}\text{x}-\text{D}\text{s}{\|}_{F}^{2}+\lambda \|\text{s}\|\;, \end{array}$$
(4) Update step for μ. By recalling that the matrixes, ${\tilde{\text{W}}}_{b}^{\;}$ and ${\tilde{\text{W}}}_{w}^{\;}$ can be built according to the obtained $\tilde{\text{Q}}$ and **S**. When $\tilde{\text{Q}}$ and $\tilde{\text{Q}}$ are learned, the solution of μ is $\frac{\partial L}{\partial \mu }=0$, and it can be obtained by a closed-form solution
  (15) $$\begin{array}{l} \mu =\frac{tr\left({\tilde{\text{Q}}}^{T}{\tilde{\text{W}}}_{b}^{\;}\tilde{\text{Q}}\right)}{2tr\left({\tilde{\text{Q}}}^{T}{\tilde{\text{W}}}_{w}^{\;}\tilde{\text{Q}}\right)}, \end{array}$$
  Based on the above analysis, the implementation process of OPFDDL is described in Algorithm 1. We initialize the sub-dictionary for each class by the K-SVD algorithm, and then, we integrate them to form the initialization dictionary **D**.
  When the projection matrix **Q** and dictionary **D** are learned, we perform the following procedure to run testing work. The testing procedure of OPFDDL is illustrated in Figure 3. For each testing EEG signal **z**, its *r*th frequency band feature is denoted as **z**^*r*^. We map **z**^*r*^ into projection subspace using **Q**^*r*^ and classify its class label according to the smallest reconstruction error on each class as follows:
  (16) $$\begin{array}{l} y^{r}=\arg\min{\left\|{\left({\text{Q}}^{r}\right)}^{T}{\text{z}}^{r}-{\text{D}}_{j}\overset{}{\underset{j}{\tilde{\text{D}}}}{\text{z}}^{r}\right\|}_{2}, \end{array}$$
  where ${\tilde{\text{D}}}_{j}^{\;}={\left({\text{D}}_{j}^{T}{\text{D}}_{j}^{\;}\right)}^{-1}{\text{D}}_{j}^{T}$ is the pseudo-inverse of dictionary ${\text{D}}_{j}^{\;}$ of class *j*.
  Finally, we use the majority voting to identify the class label of signal **z**, i.e.,
  (17) $$\begin{array}{l} y=\arg\underset{j}{\max}\Delta_{j}, \end{array}$$
  where Δ_*j*_ is the number of votes for class *j*.

**Table:** Algorithm 1 The OPFDDL algorithm

Input: Multiple frequency band EEG signals **X**^*r*^(*r* = 1, 2, ..., *R*) with their class labels. Output: Projection matrix **Q** and dictionary **D**. Initialization: Initialize **D** and **S** using the K-SVD algorithm and initialize $\tilde{\text{Q}}$ such that $\tilde{\text{Q}}{\tilde{\text{Q}}}^{T}=1$. Repeat Sparse codes update: Compute sparse code **s** for each training sample using Equation (14); Projection matrix update: Compute $\tilde{\text{Q}}$ using Equation (10); Dictionary update: Compute **D** using Equations (11–13); Adaptive weight update: Compute μ using Equation (15); Until convergence

## Experiment

### Experimental Settings

Following the study of Li Y. et al. (2019), we used extracted methods of three features on the SEED dataset, including differential entropy (DE), power spectral density (PSD), and fractal dimension (FD). We investigated the EEG features over all frequency bands per second with no overlap in each channel. We used the random 10 trials in each subject for model training and the rest 5 trials for testing. The classification performance corresponding to each period is recorded for each subject. For DREAMER dataset, to balance the number and length of the segments, we divided the 60-s EEG signals into 59 blocks with an overlap rate of 50%. The DE feature extraction method was carried out and 14-dimensional features for each frequency band were obtained. For each subject, we trained our model using the random 12 trials and the rest 6 trials for testing.

We compared our proposed model with five machine learning methods, including SVM (Cortes and Vapnik, 1995), K-SVD (Aharon et al., 2006), PCB-ICL-TSK (Ni et al., 2020b), DDL (Zhou et al., 2012), and dictionary pair learning (DPL) (Ameri et al., 2016). The Gaussian kernel and Gaussian fuzzy membership were used in SVM and PCB-ICL-TSK, respectively. The parameters in comparison methods were set according to the default settings in corresponding methods. In OPFDDL, the dimension of the projection subspace was set as 90% of the dimension of the EEG signal features. The number of atoms in each class was selected in {10, 15, 20, 25, 30, 35}. The λ parameter in Equation (2) was set as 0.01. We used the 5-fold cross-validation method to select the optimal parameters, and we performed five independent runs to evaluate the classification accuracy of all methods.

### Experiment Results on the SEED Dataset

In this subsection, we performed the comparison experiments on the SEED dataset using various combinations of frequency bands and various features. The average accuracy performances of all methods with three feature methods are summarized in Table 2. From these results, we have the following observations: (1) Under different frequency band combinations, the results of total frequency bands of all methods are the best. For example, the classification accuracy of OPFDDL using all frequency bands is 6.70, 3.83, and 2.17% higher than that using frequency bands β + γ, α + β + γ, and θ + α + β + γ. The classification accuracy of SVM using all frequency bands is 3.57, 2.90, and 1.76% higher than that using frequency bands β + γ, α + β + γ, and θ + α + β + γ. In addition, in most cases, the SDs of all methods are small in all five bands. It demonstrates that multiple bands are helpful for EEG-based ER, due to that the features of each band have discrimination ability and five bands are complementary for distinguishing EEG emotions. (2) The classification performance of the three features is comparable. The performance of the DE feature is slightly better and shows an advantage in most of the cases. The classification accuracy of OPFDDL using the DE feature is 88.87%. It indicates that the DE feature is suitable to deal with EEG emotion signals. (3) OPFDDL outperforms all comparison methods, especially in the case of all five bands. It is because that OPFDDL can effectively integrate band-independent information and inter-band correlation information. The encouraging results indicate that direct concatenation of five frequency bands of EEG data cannot well-exploit the inherent distinguishing characteristics of data. Considering the common information of multiband and band-specific information shared in each band, it is important to jointly learn multiple band representations. (4) Compared with K-SVD and ODFDL, OPFDDL generates the shared common dictionary on all frequency bands in the projected subspace, which can maintain the data structure of multiple frequency bands. In addition, based on the Fisher discrimination criterion of maximizing within-class compactness and minimizing between-class separation, OPFDDL can well learn the discriminative dictionary from the cooperation of multiple frequency bands.

**Table 2:** The average accuracies (SDs) of all methods under four combinations of frequency bands and three feature methods.

**Features**	**Methods**	**β + γ**	**α + β + γ**	**θ + α + β + γ**	**δ + θ + α + β + γ**
PSD	SVM	77.41	78.08	79.22	80.98
		(12.65)	(11.21)	(11.01)	(10.38)
	KSVD	77.03	78.00	79.46	81.28
		(12.28)	(11.76)	(11.28)	(10.47)
	PCB-ICL-TSK	77.53	80.10	80.29	81.26
		(11.76)	(11.29)	(10.63)	(10.23)
	DDL	79.79	80.85	81.78	83.16
		(10.49)	(10.56)	(10.01)	(9.45)
	DPL	80.46	81.12	82.28	83.42
		(10.58)	(10.02)	(9.21)	(8.64)
	OPFDDL	**81.00**	**83.27**	**84.93**	**87.10**
		(10.26)	(9.75)	(9.32)	(8.32)
DE	SVM	79.58	80.46	80.89	81.89
		(12.76)	(12.88)	(11.43)	(10.93)
	KSVD	78.32	78.83	80.07	82.06
		(12.77)	(12.05)	(12.11)	(10.85)
	PCB-ICL-TSK	79.66	80.55	81.36	82.49
		(11.63)	(11.09)	(10.35)	(10.04)
	DDL	80.26	81.52	83.75	84.19
		(10.56)	(10.09)	(9.43)	(9.11)
	DPL	81.76	82.12	83.37	84.34
		(10.74)	(10.12)	(9.21)	(8.65)
	OPFDDL	**82.09**	**85.34**	**86.92**	**88.87**
		(10.30)	(9.65)	(9.21)	(8.29)
FD	SVM	78.02	78.94	79.42	81.25
		(12.43)	(12.32)	(11.86)	(11.01)
	KSVD	77.56	78.18	79.79	81.61
		(12.48)	(12.02)	(11.63)	(11.12)
	PCB-ICL-TSK	78.34	80.43	80.98	81.65
		(11.57)	(11.21)	(10.68)	(9.95)
	DDL	80.21	81.23	82.46	83.64
		(10.75)	(10.12)	(9.24)	(9.15)
	DPL	80.74	81.81	82.79	84.09
		(10.35)	(10.07)	(9.31)	(9.17)
	OPFDDL	**81.94**	**83.96**	**85.61**	**87.98**
		(10.14)	(9.64)	(9.32)	(9.01)

*The highest average accuracy is shown in bold type*.

**Figure 4:**
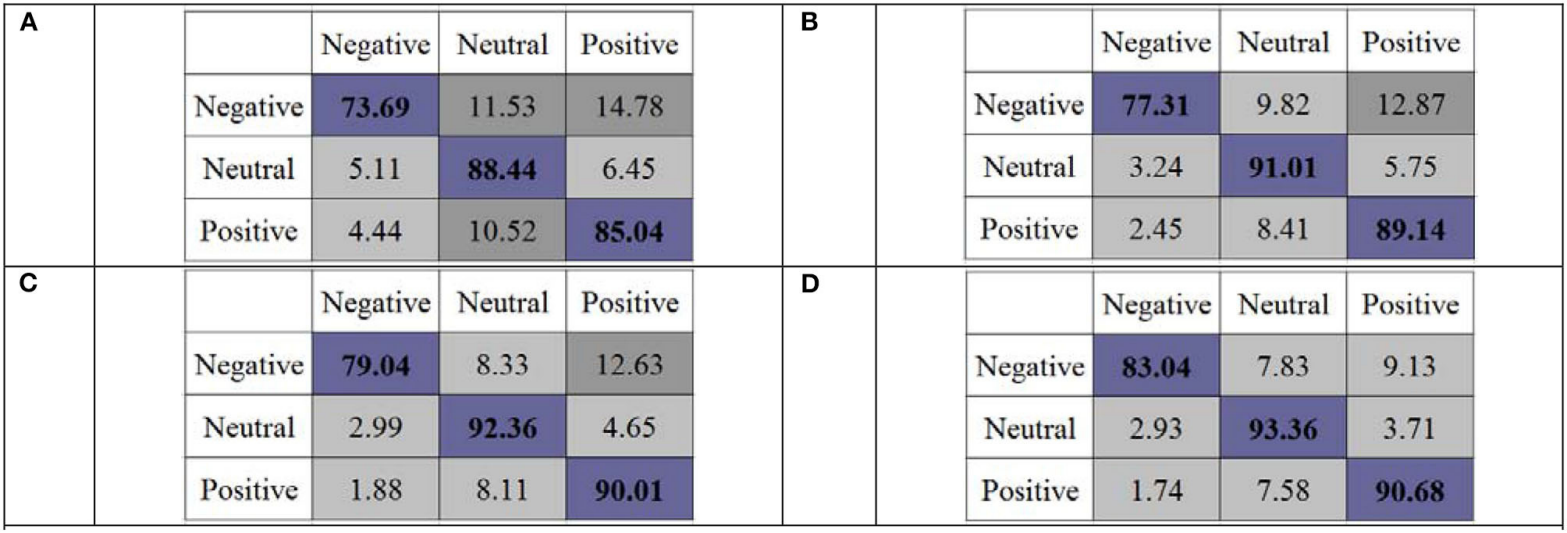
Confusion matrices of OPFDDL of frequency bands using differential entropy (DE) features. **(A)** β + γ, **(B)** α + β + γ, **(C)** θ + α + β + γ, and **(D)** δ + θ + α + β + γ.

To further validate the discrimination ability of OPFDDL, in Figure 4, we reported the confusion matrix of the OPFDDL model with the DE feature. As shown in Figure 4, OPFDDL achieves a better classification performance on positive and neutral emotions than negative emotions in all cases. The performance result of OPFDDL is similar to that in references (Zheng and Lu, 2015; Li Y. et al., 2019). This suggests that subjects may have different EEG signals when experiencing negative emotions and have similar EEG signals when experiencing positive and natural emotions. In addition, it can be seen that OPFDDL achieves the best classification performance (see Figure 4D) which uses all five frequency bands.

### Experiment Results on the DREAMER Dataset

In this subsection, we performed the comparison experiments on the DREAMER dataset. We performed the comparison experiment using the DE feature. Similar to the SEED dataset, our model is compared with the abovementioned five methods. In the experiment, we verified the performance of OPFDDL according to valence, arousal, and dominance. The accuracy performance of all methods under the frequency band θ + α + β is shown in Table 3. From the results, we can see that OPFDDL obtained the highest accuracies of 89.84, 90.11, and 89.96% in terms of arousal, valence, and dominance. Similar to the performance results on the SEED dataset, OPFDDL performed best among all comparison methods. Based on the Fisher discrimination criterion, OPFDDL can well-learn the intrinsic relationships of EEG bands and can obtain the discriminative dictionary from multiple frequency bands cooperation in the projection subspace. In addition, the joint optimization strategy, which addresses the shared projection subspace and dictionary learning, also can incrementally enhance the recognition performance of our proposed model. Thus, our proposed model can utilize more distinctive representations of multiple frequency bands of EEG signals.

**Table 3:** Average accuracies (SDs) of all methods using DE feature on DREAMER dataset.

**Methods**	**Valence**	**Arousal**	**Dominance**
SVM	86.35	86.19	86.43
	(5.21)	(5.04)	(4.89)
KSVD	86.77	86.94	87.38
	(5.28)	(5.08)	(4.89)
PCB-ICL-TSK	86.79	87.66	86.90
	(5.26)	(5.02)	(4.87)
DDL	87.54	87.85	87.99
	(5.26)	(5.00)	(4.93)
DPL	87.61	88.33	87.98
	(5.21)	(4.94)	(4.90)
OPFDDL	**89.84**	**90.11**	**89.96**
	(5.19)	(4.90)	(4.74)

*The highest average accuracy is shown in bold type*.

Then, we recorded the average accuracies of each subject for the OPFDDL model using the DE feature in terms of arousal, valence, and dominance. The experimental results are shown in Table 4. The proposed OPFDDL model had achieved satisfactory recognition performance for all three dimensions of arousal, valence, and dominance. Based on the structure of dictionary learning and the principles of projection and Fisher discrimination criterion, OPFDDL can make better use of discriminative information of different frequency band data and has stronger generalization ability, so it can be effectively used in EEG emotion classification task.

**Table 4:** Average accuracies of each subject for OPFDDL model using differential entropy (DE) feature on DREAMER dataset.

	**Valence**	**Arousal**	**Dominance**
Subject 1	93.52	93.13	95.65
Subject 2	90.45	90.08	86.04
Subject 3	86.26	85.53	81.46
Subject 4	96.87	96.32	96.84
Subject 5	93.71	94.56	95.22
Subject 6	81.05	81.04	78.04
Subject 7	84.46	84.02	82.64
Subject 8	85.42	84.97	86.73
Subject 9	81.27	82.43	85.05
Subject 10	88.95	88.04	90.43
Subject 11	82.43	83.10	87.65
Subject 12	92.48	92.15	93.04
Subject 13	89.26	92.07	90.65
Subject 14	95.19	94.66	94.86
Subject 15	98.53	98.75	98.05
Subject 16	87.16	86.78	86.43
Subject 17	87.84	89.01	88.05
Subject 18	89.45	89.02	87.21
Subject 19	94.77	95.43	94.05
Subject 20	91.01	88.03	89.23
Subject 21	93.16	94.20	94.07
Subject 22	94.83	96.04	95.11
Subject 23	88.35	93.21	92.75

### Parameter Variations

**Figure 5:**
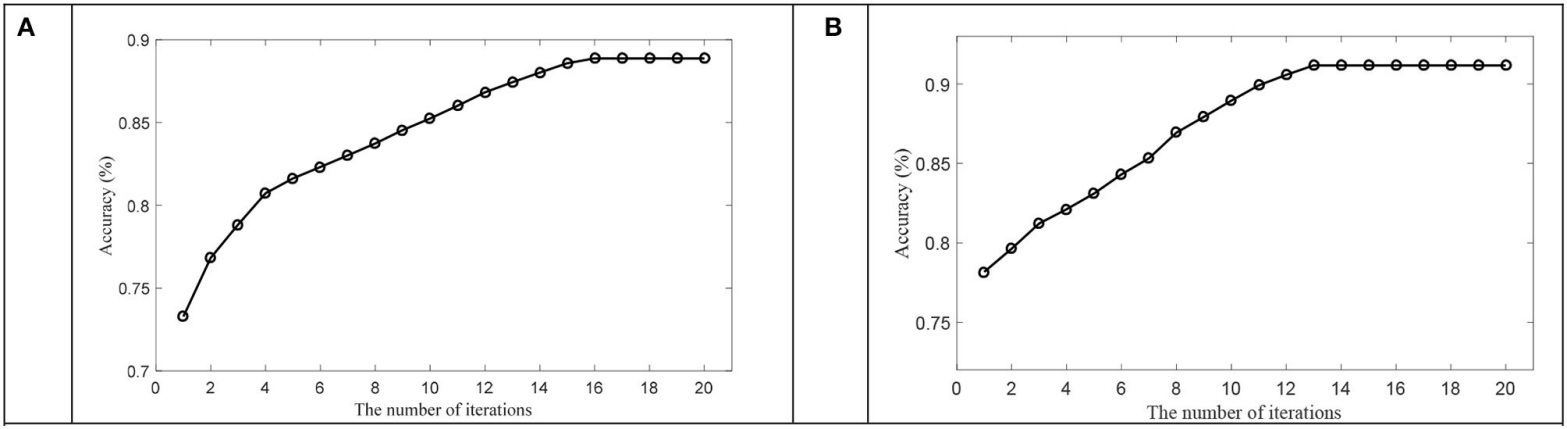
The accuracy with the number of iterations on **(A)** SEED dataset and **(B)** DREAMER dataset.

In this subsection, we first discussed the convergence of OPFDDL using the DE feature of total frequency bands on SEED and DREAMER datasets. The threshold for iteration stop was set as 10^−3^. Figure 5 plots the accuracy that varies with the number of iterations on one subject in two datasets. The results verify the convergence of OPFDDL. It can be seen that the OPFDDL model can achieve convergence within 20 iterations.

**Figure 6:**
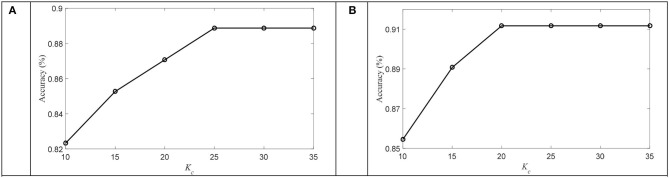
The accuracy with different *K*_*c*_ on **(A)** SEED dataset and **(B)** DREAMER dataset.

Then, we discussed the number of atoms used in OPFDDL. The number of atoms in each class *K*_*c*_ was increased from 10 to 35 in increments of 5. Figure 6 plots the accuracy that varies with the parameter *K*_*c*_. The results show that after an initial dramatic increase, the classification accuracy of OPFDDL becomes stable after *K*_*c*_ = 20. In addition, the variation trend of accuracy is consistent on two datasets. Thus, the classification performance of OPFDDL is acceptable for small dictionary sizes.

## Conclusions

Most previous machine learning methods focus on extracting feature representations for total frequency bands together without considering specific discriminative information of different frequency bands. In this study, we propose collaborative learning of multiple frequency bands for EEG-based ER. In particular, our model is an integration of projection and dictionary learning based on the Fisher discrimination criterion. For subspace projection optimization, a shared subspace is employed for each frequency band such that the band-specific representations and shared band-invariant information can be simultaneously utilized. For dictionary learning optimization, a shared dictionary is learned from the projected subspace where the Fisher discrimination criterion is used to minimize within-class sparse reconstruction error and maximize between-class sparse reconstruction error. The joint learning strategy allows the model to extend easily. Consequently, we obtain a discriminative dictionary with a small size. We have performed the experiments and proved the performance of OPFDDL on two real-world EEG emotion datasets, i.e., SEED and DREAMER. For further studies, we will try to utilize and test more discriminative sparse representation criteria in our model. In addition, we only consider subject-dependent classification in EEG emotion identification. Applying this model to subject-independent classification is a challenging work.

## Data Availability Statement

The SEED dataset analyzed for this study can be found in this link (http://bcmi.sjtu.edu.cn/~seed/seed.html). The DREAMER dataset analyzed for this study can be found in this link (https://zenodo.org/record/546113).

## Author Contributions

XG, JZho, and JZhu conceived and developed the theoretical framework of the study. All authors carried out experiment and data process, and drafted the study.

## Conflict of Interest

The authors declare that the research was conducted in the absence of any commercial or financial relationships that could be construed as a potential conflict of interest.
